# Optimal control strategies for toxoplasmosis disease transmission dynamics via harmonic mean-type incident rate

**DOI:** 10.1038/s41598-024-63263-w

**Published:** 2024-06-01

**Authors:** Usman Khan, Farhad Ali, Ohud A. Alqasem, Maysaa E. A. Elwahab, Ilyas Khan, Ariana Abdul Rahimzai

**Affiliations:** 1 Department of Mathematics, City University of Science and Information Technology, Peshawar, 25000 Khyber Pakhtunkhwa Pakistan; 2 Department of Mathematical Sciences, College of Science, Princess Nourah Bint Abdulrahman University, P.O. Box 84428, 11671 Riyadh, Saudi Arabia; 3 Department of Mathematics, College of Science Al-Zulfi, Majmaah University, 11952 Al-Majmaah, Saudi Arabia; 4 Department of Mathematics, Saveetha School of Engineering, SIMATS, Chennai, Tamil Nadu India; 5 Department of Mathematics, Education Faculty, Laghman University, Mehtarlam City, 2701 Laghman Afghanistan

**Keywords:** Toxoplasmosis transmission, Deterministic epidemic model, Stability analysis, Numerical simulation, Optimal control, Biological techniques, Biotechnology, Diseases, Engineering, Mathematics and computing

## Abstract

Toxoplasma infection in humans is considered due to direct contact with infected cats. Toxoplasma infection (an endemic disease) has the potential to affect various organs and systems (brain, eyes, heart, lungs, liver, and lymph nodes). Bilinear incidence rate and constant population (birth rate is equal to death rate) are used in the literature to explain the dynamics of Toxoplasmosis disease transmission in humans and cats. The goal of this study is to consider the mathematical model of Toxoplasma disease with harmonic mean type incident rate and also consider that the population of humans and cats is not equal (birth rate and the death rate are not equal). In examining Toxoplasma transmission dynamics in humans and cats, harmonic mean incidence rates are better than bilinear incidence rates. The disease dynamics are first schematically illustrated, and then the law of mass action is applied to obtain nonlinear ordinary differential equations (ODEs). Analysis of the boundedness, positivity, and equilibrium points of the system has been analyzed. The reproduction number is calculated using the next-generation matrix technique. The stability of disease-free and endemic equilibrium are analyzed. Sensitivity analysis is also done for reproduction number. Numerical simulation shows that the infection is spread in the population when the contact rate $\beta_{h}$ and $\beta_{c}$ increases while the infection is reduced when the recovery rate $\delta_{h}$ increases. This study investigates the impact of various optimal control strategies, such as vaccinations for the control of disease and the awareness of disease awareness, on the management of disease.

## Introduction

Basically, it is a parasite that lives inside host cells, controlling vital processes in order to acquire nutrients, evading the immune system^1^. From contaminated water, soil, or infected meat, oocysts, trachyzoites, or tissue cysts (bradyzoites) enter the host cell. Three major reservoirs for T godii are domestic and wild cats, nonliving reservoirs such as soil and water contaminated with feces, and animal reservoirs such as bradyzoites in tissue cysts. Sheep, goats, rodents, cattle, swine, chickens, and birds are intermediate hosts for T gondii. This virus is transmitted by ingestion of oocysts excreted in feline feces, for which tissue cysts are formed when exposed to litter, soil, and water. In terms of transmission to other animals, this is the most common method. In addition, oocysts from contaminated foods and raw meat can transmit T gondii. It is known as food born transmission. During pregnancy, a mother can infect her unborn child with congenital toxoplasmos (transplacental transmission). Women with acute infections during pregnancy can pass these illnesses on to their unborn children, causing mental retardation, blindness, epilepsy, and epilepsy-type illnesses. In a recent study, it was reported that dogs may transmit T gondii^2^. Several treatment options exist for toxoplasmosis, including sulfonamides against murine toxoplasmosis, pyrimethamine and combined therapy in human toxoplasmosis, spiramycin during pregnancy for parasite prevention, and clindamycin for individuals allergic to sulphonamides^1^.

A single-celled parasite, T. gondii is an apicomplexan parasite. Warm-blooded animals, including humans, are susceptible to this parasite. The asexual and sexual stages of its life cycle make T. gondii an extremely versatile pathogen capable of infecting almost every nucleated cell in the body. This parasite possesses a unique life cycle characterized by indefinite replication through sexual and asexual subcycles. Animals with warm blood, especially cats and humans, can undergo the asexual cycle of Toxoplasma^3^. The symptoms of Toxoplasma gondii infection can vary depending on the individual's immune status and the stage of infection. In immunocompetent individuals, fever, sore throat, swollen lymph nodes, headache, muscle ache, fatigue, and a sore throat, and body rash are common symptoms of the infection. In humans, Toxoplasma gondii infection typically takes five to 23 days to develop after exposure^4^. Individuals' immune status, parasite dose, and route of infection can affect the incubation period^5^. The presence of more than 20 million oocysts excreted by cats can cause infections in humans within 4 to 13 days after infection with Toxoplasma^6^. Vertical transmission of T. gondii can occur through the transfer of tachyzoites from the mother to the fetus via the placenta^7^. Toxoplasma reproduces rapidly and slowly in asexual tissues like the brain and heart. A carnivorous or scavenging animal can transmit the disease by consuming bradyzoite-infected tissue^8^. It is also possible to accidentally transmit diseases through contaminated feed ^5^. Domestication of animals by humans has been a part of history for over 12,000 years, and cats have been domesticated for 4000 years. The number of cats owned in western countries is on its way to surpassing those of dogs. According to the Pet Food Institute in Washington, DC, there are 70.2 million cats in the U.S, while there are 5.5 million in Spain, and there are 10% of households in Colombia with cats^9^. Cats are considered a transmission vector for Toxoplasmosis, similar to how Aedes aegypti mosquitoes are associated with the transmission of dengue fever^10^. In certain small islands where cats are absent but other animals are present, the pervasiveness of Toxoplasmosis is negligible^8^. Infected cats' feces can also contaminate food and vegetables. In tropical countries, the most common route of infection is oral ingestion, which causes Toxoplasma gondii infection^11^. Different species of animals have varying levels of antibodies to Toxoplasma gondii, but pigs have a range of 26% to 78%. Argentine, Brazilian, and Colombian variations illustrate the difference among Latin American countries^12^. Human populations are becoming increasingly infected with Toxoplasma gondii according to several studies^13^.

In recent years, mathematical models have been used to study epidemics involving parasites, viruses, and, more recently, COVID-19. It is true that mathematical models can simplify the real world in many fields, but they are still be useful sources of insight for a wide range of real processes. A wide variety of diseases have been studied using mathematical models^14–16^. T gondii infection dynamics have been explored using multiple mathematical models. Toxoplasmosis disease dynamics was studied in Colombia using a mathematical model^17^. Simulations of the model using available data show that some (hygiene actions, education programs, more testing and treatments) strategies can control toxoplasmosis parasites. Toxoplasmosis transmission in human and cat populations with constant population (birth rate is equal to death rate) has been investigated^7^. Moreover, fractional order derivatives have been used to study Toxoplasmosis transmission in humans and cats (birth rate equals death rate)^18^. A mathematical model is developed to predict Toxoplasmosis transmission in cat populations and evaluate the effectiveness of vaccination strategies^19^. In order to understand how Toxoplasmosis disease behaves in populations of different sizes, a mathematical model was used, providing insight for disease control and management^17^. A mathematical model is developed that characterizes the transfer of Toxoplasma gondii between cats and the environment, shedding light on the dynamics of transmission^20^. In^21^, Toxoplasmosis dynamics is studied using a mathematical model incorporating multiple hosts, vertical transmission, and cat vaccinations. The model provides valuable insights for the formulation of effective disease control strategies. The authors in^22^ examined stability analysis and optimal control strategies for an epidemic model such as vaccination and treatment. In^23^ the authors investigated the effect of government policy, public response and social behavioral reaction on the disease dynamics. The also analyzed the effects of environmental fluctuations and time-dependent control strategies on the disease dynamics. The authors have show that Numerical figure depicts that the governmental action plays a crucial role to control an epidemic situation, and the system turns out to be disease-free sooner if the government takes action at an early stage during a disease outbreak. A SIVIS model was used by the authors in^24^ to examine epidemic dynamics, focusing on factors including heterogeneous susceptibility, government interventions, social behaviors, and vaccination effects. In addition to exploring time-dependent strategies for managing social behaviors and pharmaceutical treatments, they analyzed the effects of periodic transmission rates on disease dynamics, framing the problem as an optimal control problem. By taking a societal and environmental perspective, the authors in^25^ explored the dynamics of transmission of diseases through asymptomatic carriers of the disease. The authors in^26^ investigated the transmission dynamics of Gonorrhea in a structured population and examined the impact of different optimal control measures, including education, condom use, vaccinations, and treatment. In^27^ the authors used a non-autonomous nonlinear model to study the cost effectiveness of COVID-19 control interventions. In order to determine their effectiveness and cost-saving potential, they proposed and evaluated fourteen optimal control strategies, including social distancing, hygiene practices, and fumigation. Its numerical simulation shows that strategy 1 (practicing physical and social distancing protocols) is the most effective and cost-saving control intervention in Saudi Arabia without vaccination. In^28^, the authors examined non-seasonal and seasonal relapse models for Q fever disease and evaluated the cost-effectiveness of different control strategies, including animal separation, diagnosis-treatment, and disinfection. The authors studied the transmission potential of Zika virus with the impact different optimal control strategies such as bed nets and mosquito repellents, treatment of Zika patients, and the spray of insecticides on mosquitoes in ^29^. The authors in^30^ studied the transmission dynamics of Q fever by using non-fractional and fractional mathematical model.

In both human and cat populations, mathematical modeling is useful for studying Toxoplasmosis dynamics. To explore the disease dynamics, researchers employ an epidemiological model that considers transmission dynamics in both populations. In a study^7^, the author examined the transmission dynamics of Toxoplasmosis in humans and cats population, considering a bilinear incident rate and a constant population. Based on the existing literature, the dynamics of Toxoplasmosis disease in human and cat population with the Harmonic mean type incident rate and different optimal control strategies have not been studied yet. Therefore, this article aims to do such an attempt. As motivated from the above literature, the aim of this study is to consider a harmonic mean type of incidence rate is used between susceptible humans and infected vectors in order to have a rapid extinction of the infected population. The mean of two values indicates the centrality of a dataset. A geometric mean is mainly used to average ratios or changes in data. On other hand Harmonic mean is less sensitive to large values than arithmetic or geometric means and are sometimes used for highly skewed data. In comparison to other incidence rates, the harmonic incidence rate indicates the possibility of the population approaching extinction in a finite amount of time, although more quickly. When defining the number of persons in respect to the same unit, the harmonic mean is a superior incidence rate than the others. Furthermore, the harmonic incidence rate frequently offers a correct average of the ratios and rates. It is especially vulnerable to one number that is below normal. Moreover, different strategies for optimal control are presented, including vaccines for diseases and awareness/education of the population about them. The rest od paper is arranged as; the mathematical model of Toxoplasmosis Positivity, boundedness, disease free equilibrim, reproduction nimber and endemic equilibrium of Toxoplasmosis model is investigated in Section “Model formulation”. Section “Local stability of disease-free equilibria” shows the local stability of disease free and endemic equilibrium. Section “Global stability of disease-free equilibria” shows the globle stability of disease free and endemic equilibrium. In Section “Numerical simulation” the sensitivity analysis of Toxoplamosis is presented. Numerical simulations of Toxomplasmosis and its result and discussion were given in Sections “Results and discussion” & “Optimal control model of toxoplasmosis”. The optimal control model of Toxoplosmosis and its numerical simulation were is given in Section “Optimal control model of toxoplasmosis”. The conclusions are covered in the final Section “Conclusion”.

## Model formulation

**Figure 1:**
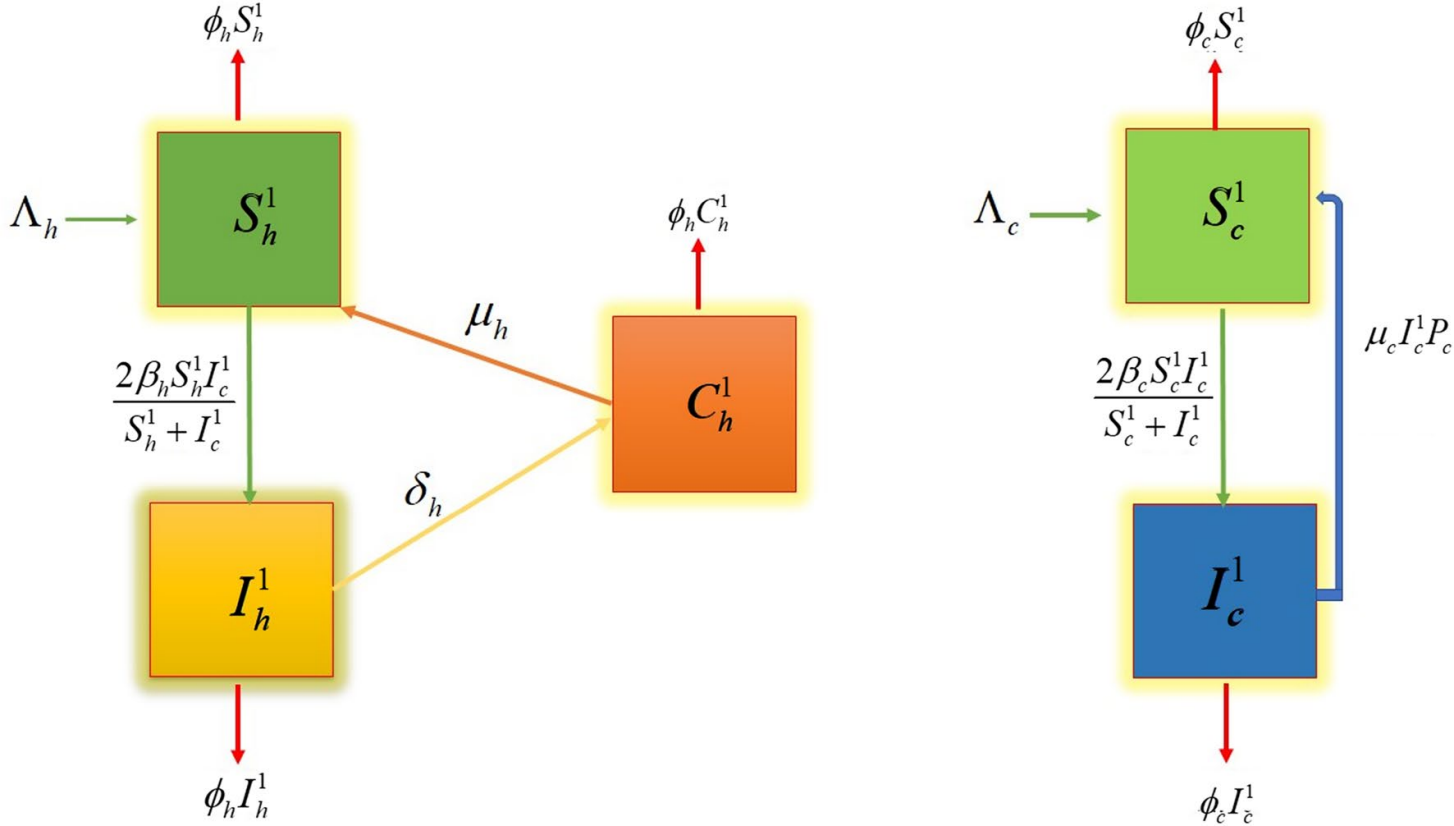
Flow chart of the transmission dynamics of Psittacosis disease.

Figure 1 illustrates a generalized model for Toxoplasmosis transmission dynamics in human and cat populations based on features of virus transmission. $N_{h} (t)$ represents total population of human and it is divided into three compartments by their disease status, as follows: susceptible, infected and recovered classes and is represented by $S_{h}^{1} (t)$, $I_{h}^{1} (t)$ and $C_{h}^{1} (t)$ respectively at any time $\psi_{{h_{\,1} }}$ $N_{c} (t)$ represents total population of cats, and it is further subdivided into $S_{c}^{1} (t)$ and $I_{c}^{1} (t)$ respectively denote susceptible and infected cats population at any time $\Lambda_{{p\,_{1} }}$ All the variables and parameters elaborated in the above system are positive.

1 $$\begin{gathered} \frac{{dS_{h}^{1} }}{dt} = \Lambda_{h} + \mu_{h} C_{h}^{1} - \frac{{2\beta_{h} S_{h}^{1} I_{c}^{1} }}{{S_{h}^{1} + I_{c}^{1} }} - \phi_{h} S_{h}^{1} , \hfill \\ \frac{{dI_{h}^{1} }}{dt} = \frac{{2\beta_{h} S_{h}^{1} I_{c}^{1} }}{{S_{h}^{1} + I_{c}^{1} }} - \delta_{h} I_{h}^{1} - \phi_{h} I_{h}^{1} , \hfill \\ \frac{{dC_{h}^{1} }}{dt} = \delta_{h} I_{h}^{1} - \mu_{h} C_{h}^{1} - \phi_{h} C_{h}^{1} , \hfill \\ \frac{{dS_{c}^{1} }}{dt} = \Lambda_{c} + \mu_{c} I_{c}^{1} P_{c} - \frac{{2\beta_{c} S_{c}^{1} I_{c}^{1} }}{{S_{c}^{1} + I_{c}^{1} }} - \phi_{c} S_{c}^{1} , \hfill \\ \frac{{dI_{c}^{1} }}{dt} = \frac{{2\beta_{c} S_{c}^{1} I_{c}^{1} }}{{S_{c}^{1} + I_{c}^{1} }} - \mu_{c} I_{c}^{1} P_{c}^{{}} - \phi_{c} I_{c}^{1} . \hfill \\ \end{gathered}$$

Initial conditions:

2 $$S_{h}^{1} \left( 0 \right) \ge 0,\,\,I_{h}^{1} \left( 0 \right) \ge 0,\,C_{h}^{1} \left( 0 \right) \ge 0,\,S_{c}^{1} \left( 0 \right) \ge 0,\,I_{c}^{1} \left( 0 \right) \ge 0.$$

The parameters in above system of equations are defined Table 1 whereas the flow chart of the transmission dynamics of Psittacosis disease is shown in Fig. 1.

**Table 1:** Parameters and their description.

Parameters	Description	Dimension/unit
$\Lambda_{h}$	Recruitment rate of humans	Population/T
$\mu_{h}$	Partial recovery	Population/T
$\beta_{1h}$	Contact rate	Population/T
$\phi_{h}$	Natural mortality rate of humans	Population/T
$\delta_{\,h}$	Recovery rate of humans	Population/T
$\Lambda_{\,c}$	Recruitment rate of cats	Population/T
$\mu_{\,c}$	Partial recovery of cats	Population/T
$\beta_{c}$	Contact rate of infected cats	Population/T
$\phi_{\,c}$	Natural mortality rate of cats	Population/T
$P_{\,c}$	Probability of a susceptible cat being born from an infected one	Dimensionless

### Positive invariant set

Model system (1) solutions are uniformly bounded in a proper subset $\Lambda \in R^{5}$^31^.

Let the human population at time t.:

At any time t the human population as follow:

3 $$N_{h} (t) = S_{h}^{1} (t) + I_{h}^{1} (t) + C_{h}^{1} (t)$$

For the human population in model (1), the feasible solution set will be:

4 $$k_{h} = \left\{ {(S_{h}^{1} ,\,I_{h}^{1} ,\,C_{h}^{1} ) \in R^{3} ,\,0 \le N_{h} \le \frac{{\Lambda_{\,h} }}{{\phi_{\,h} }}} \right\}$$

Similarly consider the cat population at any time *t*:

5 $$N_{c} = S_{c}^{1} (t) + I_{c}^{1} (t)$$

The feasible solution set of mosquitoes and ticks population in the model (1) will be:

6 $$k_{c} = \left\{ {(S_{c}^{1} ,\,I_{c}^{1} ) \in R^{2} ,\,0 \le N_{c} \le \frac{{\Lambda_{\,c} }}{{\phi_{\,c} }}} \right\}.$$

Therefore, the feasible solution of Eq. (1) is:

7 $$k = \left\{ {\left( {S_{h}^{1} ,\,I_{h}^{1} ,\,C_{h}^{1} ,\,S_{c}^{1} ,\,I_{c}^{1} } \right) \in R^{3} \times R^{2} = R^{5} ,\,0 \le N_{h} \le \frac{{\Lambda_{\,h} }}{{\phi_{\,h} }},0 \le N_{c} \le \frac{{\Lambda_{\,c} }}{{\phi_{\,c} }}} \right\}.$$

### Diseases free equilibria

The infection-free (DFE) point of the system of Eq. (1) is found by substituting $I_{h}^{1} = I_{c}^{1} = 0$ into the system (1) ^32^.

8 $$DFE = \left\{ {\frac{{\Lambda_{\,h} }}{{\phi_{\,h} }},0,0,\frac{{\Lambda_{\,c} }}{{\phi_{\,c} }},0} \right\}.$$

### Reproduction number ($R_{0}$)

In order to understand how diseases spread and how to control them, it is important to determine the reproduction number. It serves as an indicator of both disease spread and control. When the $R_{0} < 1$, the disease-free equilibrium is stable, and the disease is eliminated from the population, both locally and globally. This helps prevent the outbreak of epidemics. On the other hand, when $R_{0} > 1$, the local and global endemic equilibrium is stable. The value of $R_{0}$ in the model (1) can be obtained using the next-generation matrix method^16^.

9 $$R_{0} = \frac{{2\beta_{\,c} }}{{P_{\,c} \mu_{\,c} + \phi_{\,c} }}.$$

### Endemic equilibria

Endemic equilibrium $E^{*}$, means the disease exists in a population for a long time. Model system (1) is rearranged to get $S_{h}^{1*} ,R_{h}^{1*} ,S_{c}^{1*}$ and $I_{c}^{1*}$ in terms of $I_{h}^{1*}$^33^. Thus,

10 $$\left. \begin{gathered} S_{h}^{1*} = \frac{{\Lambda_{\,h} \mu_{\,h} - I_{h}^{1} \delta_{\,h} \phi_{\,h} + \Lambda_{\,h} \phi_{\,h} - I_{h}^{1} \mu_{\,h} \phi_{\,h} - I_{h}^{1} \phi_{\,h}^{2} }}{{\phi_{\,h} \left( {\mu_{\,h} + \phi_{\,h} } \right)}}, \hfill \\ C_{h}^{1*} = \frac{{I_{h}^{1} \delta_{\,h} }}{{\mu_{\,h} + \phi_{\,h} }}, \hfill \\ S_{c}^{1*} = \frac{{\Lambda_{\,c} \left( {\phi_{\,c} + P_{\,c} \mu_{\,c} } \right)}}{{2\beta_{\,c} \phi_{\,c} }}, \hfill \\ I_{c}^{1*} = \frac{{\Lambda_{\,c} \left( {2\beta_{\,c} - \phi_{\,c} - P_{\,c} \mu_{\,c} } \right)}}{{2\,\phi_{\,c} \beta_{\,c} }}. \hfill \\ \end{gathered} \right\}.$$

## Local stability of disease-free equilibria

### Theorem:

*When the reproduction number is less than one, the DFE is asymptotically stable, otherwise it becomes unstable* ^34^ *.*

### Proof:

As a first step, we calculated the Jacobian matrix (1) and calculated local stability of DFE as follows:

11 $$J(DFE) = \left( {\begin{array}{*{20}c} { - \phi_{\,h} } & 0 & {\mu_{\,h} } & 0 & { - 2\beta_{\,h} } \\ 0 & { - \phi_{\,h} - \delta_{\,h} } & 0 & 0 & {2\beta_{\,h} } \\ 0 & {\delta_{\,h} } & { - \phi_{\,h} - \mu_{\,h} } & 0 & 0 \\ 0 & 0 & 0 & { - \phi_{\,c} } & { - 2\beta_{\,c} } \\ 0 & 0 & 0 & 0 & {2\,\beta_{\,c} - \phi_{\,c} - P_{\,c} \mu_{\,c} } \\ \end{array} } \right)$$

Thus the eigenvalues of $J(DFE)$ are

12 $$\begin{gathered} \lambda_{1} = - \phi_{h} ,\;\lambda_{4} = - \phi_{c} , \hfill \\ \lambda_{2} = - (\delta_{h} + \phi_{h} ),\;\lambda_{5} = (P_{c} \mu_{c} + \phi_{c} )(R_{0} - 1). \hfill \\ \lambda_{3} = - (\mu_{h} + \phi_{h} ), \hfill \\ \end{gathered}$$

Since $\lambda_{\,1} ,\lambda_{\,2} ,\lambda_{\,3} ,\lambda_{\,4}$ are negative and the remaining $\lambda_{\,5}$ will be negative if $R_{\,0} < 1$ thus DFE is LAS.

### Local stability analysis of endemic equilibria

#### Theorem:

*whenever *$R_{0} > 1$ $E^{*}$* is an asymptotically steady endemic equilibria in model* (1)^35^.

#### Proof:

In order to determine local stability, we first determine the Jacobian matrix $E^{*}$ using the differential Eq. (1).

13 $$J = \left( {\begin{array}{*{20}c} {\frac{{2I_{c}^{1} S_{h}^{1} \beta_{\,h} }}{{\left( {I_{c}^{1} + S_{h}^{1} } \right)^{2} }} - \frac{{2I_{c}^{1} \beta_{\,h} }}{{I_{c}^{1} + S_{h}^{1} }} - \phi_{\,h} } & 0 & {\mu_{\,h} } & 0 & {\frac{{2I_{c}^{1} S_{h}^{1} \beta_{\,h} }}{{\left( {I_{c}^{1} + S_{h}^{1} } \right)^{2} }} - \frac{{2S_{h}^{1} \beta_{\,h} }}{{I_{c}^{1} + S_{h}^{1} }}} \\ { - \frac{{2I_{c}^{1} S_{h}^{1} \beta_{\,h} }}{{\left( {I_{c}^{1} + S_{h}^{1} } \right)^{2} }} + \frac{{2I_{c}^{1} \beta_{\,h} }}{{I_{c}^{1} + S_{h}^{1} }}} & { - \delta_{\,h} - \phi_{\,h} } & 0 & 0 & { - \frac{{2I_{c}^{1} S_{h}^{1} \beta_{\,h} }}{{\left( {I_{c}^{1} + S_{h}^{1} } \right)^{2} }} + \frac{{2S_{h}^{1} \beta_{\,h} }}{{I_{c}^{1} + S_{h}^{1} }}} \\ 0 & {\delta_{\,h} } & { - \mu_{\,h} - \phi_{\,h} } & 0 & 0 \\ 0 & 0 & 0 & {\frac{{2I_{c}^{1} S_{c}^{1} \beta_{\,c} }}{{\left( {I_{c}^{1} + S_{c}^{1} } \right)^{2} }} - \frac{{2I_{c}^{1} \beta_{\,c} }}{{I_{c}^{1} + S_{c}^{1} }} - \phi_{\,c} } & {\frac{{2I_{c}^{1} S_{c}^{1} \beta_{\,c} }}{{\left( {I_{c}^{1} + S_{c}^{1} } \right)^{2} }} - \frac{{2S_{c}^{1} \beta_{\,c} }}{{I_{c}^{1} + S_{c}^{1} }} + P_{\,c} \mu_{\,c} } \\ 0 & 0 & 0 & { - \frac{{2I_{c}^{1} S_{c}^{1} \beta_{\,c} }}{{\left( {I_{c}^{1} + S_{c}^{1} } \right)^{2} }} + \frac{{2I_{c}^{1} \beta_{\,c} }}{{I_{c}^{1} + S_{c}^{1} }}} & { - \frac{{2I_{c}^{1} S_{c}^{1} \beta_{\,c} }}{{\left( {I_{c}^{1} + S_{c}^{1} } \right)^{2} }} + \frac{{2S_{c}^{1} \beta_{\,c} }}{{I_{c}^{1} + S_{c}^{1} }} - P_{\,c} \mu_{\,c} - \phi_{\,c} } \\ \end{array} } \right),$$

14 $$J(E^{*} ) = \left( {\begin{array}{*{20}c} {P_{1} } & 0 & \tau & 0 & { - P_{7} } \\ {P_{6} } & {P_{2} } & 0 & 0 & {P_{7} } \\ 0 & \eta & {P_{3} } & 0 & 0 \\ 0 & 0 & 0 & {P_{4} } & { - P_{8} } \\ 0 & 0 & 0 & {P_{8} } & {P_{5} } \\ \end{array} } \right),$$

where

15 $$\begin{gathered} P_{1} = \frac{{2I_{c}^{1} S_{h}^{1} \beta_{\,h} }}{{\left( {I_{c}^{1} + S_{h}^{1} } \right)^{2} }} - \frac{{2I_{c}^{1} \beta_{\,h} }}{{I_{c}^{1} + S_{h}^{1} }} - \phi_{\,h} ,\;P_{4} = \frac{{2I_{c}^{1} S_{c}^{1} \beta_{\,c} }}{{\left( {I_{c}^{1} + S_{c}^{1} } \right)^{2} }} - \frac{{2I_{c}^{1} \beta_{\,c} }}{{I_{c}^{1} + S_{c}^{1} }} - \phi_{\,c} , \hfill \\ P_{2} = - \delta_{\,h} - \phi_{\,h} ,\;P_{5} = - \frac{{2I_{c}^{1} S_{c}^{1} \beta_{\,c} }}{{\left( {I_{c}^{1} + S_{c}^{1} } \right)^{2} }} + \frac{{2S_{c}^{1} \beta_{\,c} }}{{I_{c}^{1} + S_{c}^{1} }} - P_{\,c} \mu_{\,c} - \phi_{\,c} . \hfill \\ P_{3} = - \mu_{\,h} - \phi_{\,h} , \hfill \\ \end{gathered}$$

16 $$\left| {J(E^{*} ) - \lambda } \right| = \left| {\begin{array}{*{20}c} {P_{1} - \lambda_{1} } & 0 & \tau & 0 & { - P_{7} } \\ {P_{6} } & {P_{2} - \lambda_{2} } & 0 & 0 & {P_{7} } \\ 0 & \eta & {P_{3} - \lambda_{3} } & 0 & 0 \\ 0 & 0 & 0 & {P_{4} - \lambda_{4} } & { - P_{8} } \\ 0 & 0 & 0 & {P_{8} } & {P_{5} - \lambda_{5} } \\ \end{array} } \right| = 0.$$

From the above we get.

17 $$\lambda^{5} + y_{1} \,\lambda^{4} + y_{2} \,\lambda^{3} \, + \,y_{3} \,\lambda^{2} + y_{2} \,\lambda + y_{5} = 0$$

Routh–Hurwitz criterion is used to determine the remaining values of $\lambda$. The coefficients of the polynomial and the values derived from them can be arranged in the following manner to determine the exact number of roots with real parts:

18 $$z_{0} \lambda^{n} + z_{1} \lambda^{n - 1} + ... + z_{n - 1} \lambda + p_{n} = 0.$$

$$\begin{array}{*{20}c} {\lambda^{5} } & 1 & {r_{2} } & {r_{4} } \\ {\lambda^{4} } & {r_{1} } & {r_{3} } & {r_{5} } \\ {\lambda^{3} } & {a_{1} } & {a_{2} } & 0 \\ {\lambda^{2} } & {b_{1} } & {b_{2} } & \; \\ {\lambda^{1} } & {c_{1} } & 0 & \; \\ {\lambda^{0} } & {d_{1} } & \; & \; \\ \end{array}$$

where

$$\begin{aligned} & a_{1} = \frac{1}{{r_{1} }}(r_{1} r_{2} - r_{3} ),\;a_{2} = \frac{1}{{r_{1} }}(r_{1} r_{4} - r_{5} ),\;b_{1} = \frac{1}{{a_{1} }}(a_{1} r_{3} - r_{1} a_{2} ), \\ & b_{2} = r_{5} ,\;c_{1} = \frac{1}{{b_{1} }}(a_{2} b_{1} - a_{1} b_{2} ),\;d_{1} = r_{5} . \\ \end{aligned}$$

The Routh–Hurwitz criteria states that an Eigenvalue of the characteristic equation must satisfy the following condition:

19 $$\begin{gathered} r_{1} > 0,\,\,r_{2} > 0,\,\,r_{3} > 0,\,\,r_{4} > 0,\,\,r_{5} > 0,a_{1} > 0,\,\,a_{2} > 0,\,\,\,b_{1} > 0,\,\,b_{2} > 0, \hfill \\ c_{1} > 0,\,d_{1} > 0. \hfill \\ \end{gathered}$$

So $R_{0} > 1$, this means that the endemic equilibrium point is locally asymptotical.

## Global stability of disease-free equilibria

Castillo–Chavez approach is used to find the global equilibrium point free of disease.According to this method, the proposed model (1) is divided into two subsystems:

20 $$\left. \begin{gathered} \frac{{d\ell_{1} }}{dt} = G(\ell_{1} ,\ell_{2} ), \hfill \\ \frac{{d\ell_{2} }}{dt} = H(\ell_{1} ,\ell_{2} ). \hfill \\ \end{gathered} \right\}.$$

where $\ell_{1}$ and $\ell_{2}$ represent uninfected and infected individuals, respectively, that is, $\ell_{1} = (S_{h}^{1} ,C_{h}^{1} ,S_{c}^{1} ) \in R^{3}$ and $\ell_{2} = (I_{h}^{1} ,I_{c}^{1} ) \in R^{2}$. $E^{0}$ represents disease free equilibrium and define as $E^{0} = \left( {\ell_{0} ,0} \right)$. Hence, global stability at disease-free equilibrium depends on two conditions:

$\frac{{d\ell_{1} }}{dt} = G(\ell_{1} ,0)$, $x_{1}^{0}$ is globally asymptotically stable.

1. $$H(\ell_{1} ,\ell_{2} ) = B\ell_{2} - \overline{H}(\ell_{1} ,\ell_{2} )$$,where $$\overline{H}(\ell_{1} ,\ell_{2} ) \ge 0$$ for $$(\ell_{1} ,\ell_{2} ) \in k$$.
2. In the second condition, $B = D_{{\ell_{2} }} H(\ell_{1}^{0} ,0)$ is an M-matrix with positive diagonal entries, and $k$ is the feasible region.

### Lemma 2:

*If *$R_{0} < 1$*, then the equilibrium point *$E^{0} = (\ell_{1}^{0} ,0)$* of the system (1) is said to be globally asymptotically stable, if the above conditions are satisfied*^36^.

### Theorem:

*According to the proposed model (1), if *$R_{0} < 1$*, it is globally asymptotically stable at disease-free equilibrium *$E^{0}$*, and otherwise it is unstable*.

### Proof:

Proof Let $\ell_{1} = \left( {S_{h}^{1} ,C_{h}^{1} ,S_{c}^{1} } \right)$ and $\ell_{2} = \left( {I_{h}^{1} ,I_{c}^{1} } \right)$ and define $E^{0} = \left( {\ell_{0} ,0} \right)$, where

$$\ell_{1}^{0} = \left( {\frac{{\Lambda_{\,h} }}{{\phi_{\,h} }},\frac{{\Lambda_{\,c} }}{{\phi_{\,c} }}} \right)$$

By using model system (1), we have

$$\frac{{d\ell_{1} }}{dt} = G\left( {\ell_{1} ,\ell_{2} } \right),$$

21 $$\frac{{d\ell_{1} }}{dt} = \left( {\begin{array}{*{20}c} {\Lambda_{\,h} + \mu_{\,h} C_{h}^{1} - \frac{{2\beta_{\,h} S_{h}^{1} I_{c}^{1} }}{{S_{h}^{1} + I_{c}^{1} }} - \phi_{\,h} S_{h}^{1} } \\ {\delta_{\,h} I_{h}^{1} - \mu_{\,h} C_{h}^{1} - \phi_{\,h} C_{h}^{1} } \\ {\Lambda_{\,c} + \mu_{\,c} I_{c}^{1} P_{\,c} - \frac{{2\beta_{\,c} S_{c}^{1} I_{c}^{1} }}{{S_{c}^{1} + I_{c}^{1} }} - \phi_{\,c} S_{c}^{1} } \\ \end{array} } \right),$$

For $S_{h}^{1} = \left( {S_{h}^{1} } \right)^{0} ,S_{c} = \left( {S_{c}^{1} } \right)^{0}$ and $G\left( {\ell_{1} ,0} \right) = 0.$ we get

22 $$G\left( {\ell_{1} ,0} \right) = \left( {\begin{array}{*{20}c} {\Lambda_{\,h} - \phi_{\,h} S_{h}^{1} } \\ {\Lambda_{\,c} - \phi_{\,c} S_{c}^{1} } \\ \end{array} } \right) = 0.$$

From above equation as $t \to \infty ,\ell_{1} \to \ell_{1}^{0} .$ So $x_{1} = x_{1}^{0}$ is globally asymptotically stable. Now

23 $$B\left( {\ell_{2} } \right) - \overline{H}\left( {\ell_{1} ,\ell_{2} } \right) = \left( {\begin{array}{*{20}c} { - \delta_{\,h} - \phi_{\,h} } & {2\beta_{\,h} \left( {S_{h}^{1} } \right)^{0} } \\ 0 & {2\beta_{\,c} \left( {S_{c}^{1} } \right)^{0} - P_{\,c} \mu_{\,c} - \phi_{\,c} } \\ \end{array} } \right)\left( {\begin{array}{*{20}c} {I_{h}^{1} } \\ {I_{c}^{1} } \\ \end{array} } \right) - \left( {\begin{array}{*{20}c} {2\beta_{\,h} \left( {S_{h}^{1} } \right)^{0} I_{c}^{1} - \frac{{2\beta_{\,h} S_{h}^{1} I_{c}^{1} }}{{S_{h}^{1} + I_{c}^{1} }}} \\ {2\beta_{\,c} \left( {S_{c}^{1} } \right)^{0} I_{c}^{1} - \frac{{2\beta_{\,c} S_{c}^{1} I_{c}^{1} }}{{S_{c}^{1} + I_{c}^{1} }}} \\ \end{array} } \right)$$

As $2\beta_{\,h} \left( {S_{h}^{1} } \right)^{0} I_{c}^{1} \ge \frac{{2\beta_{\,h} S_{h}^{1} I_{c}^{1} }}{{S_{h}^{1} + I_{c}^{1} }}$ and $2\beta_{\,c} \left( {S_{c}^{1} } \right)^{0} I_{c}^{1} - \frac{{2\beta_{\,c} S_{c}^{1} I_{c}^{1} }}{{S_{c}^{1} + I_{c}^{1} }}$. Hence $H\left( {\ell_{1} ,\ell_{2} } \right) \ge 0$. B is clearly an M-matrix. Therefore, Lemma 2 indicates that the equilibrium point for disease-free equilibrium is globally asymptotically stable.

### Global stability analysis of endemic equilibria

When a system has global stability, it indicates that it may be started in any position and yet return to its original state of equilibrium.

#### Theorem:

*If *$R_{0} > 1$*,*$E^{*}$* the asymptotic behavior of the model (1) is globally asymptotical*^37^.

#### Proof:

With the help of the following Lyapunov function, we can calculate $E^{*}$ global asymptotic stability:

24 $$\begin{aligned} L(S_{h}^{1*} ,I_{h}^{1*} ,C_{h}^{1*} ,S_{c}^{1*} ,I_{c}^{1*} ) = & \left( {S_{h}^{1} - S_{h}^{1*} - S_{h}^{1*} \ln \frac{{S_{h}^{1} }}{{S_{h}^{1*} }}} \right) + \left( {I_{h}^{1} - I_{h}^{1*} - I_{h}^{1*} \ln \frac{{I_{h}^{1} }}{{I_{h}^{1*} }}} \right) + \left( {C_{h}^{1} - C_{h}^{1*} - C_{h}^{1*} \ln \frac{{C_{h}^{1} }}{{C_{h}^{1*} }}} \right) \\ & + \left( {S_{c}^{1} - S_{c}^{1*} - S_{c}^{1*} \ln \frac{{S_{c}^{1} }}{{S_{c}^{1*} }}} \right) + \,\,\left( {I_{c}^{1} - \,\,I_{c}^{1*} - \,\,I_{c}^{1*} \ln \frac{{I_{c}^{1} }}{{I_{c}^{1*} }}} \right). \\ \end{aligned}$$

Calculating the derivative of L directly along solution (1), we have:

25 $$\frac{dL}{{dt}} = \left( {1 - \frac{{S_{h}^{1*} }}{{S_{h}^{1} }}} \right)\frac{{dS_{h}^{1} }}{dt} + \left( {1 - \frac{{I_{h}^{1*} }}{{I_{h}^{1} }}} \right)\frac{{dI_{h}^{1} }}{dt} + \left( {1 - \frac{{C_{h}^{1*} }}{{C_{h}^{1} }}} \right)\frac{{dC_{h}^{1} }}{dt} + \left( {1 - \frac{{S_{c}^{1*} }}{{S_{c}^{1} }}} \right)\frac{{dS_{c}^{1} }}{dt} + \left( {1 - \frac{{I_{c}^{1*} }}{{I_{c}^{1} }}} \right)\frac{{dI_{c}^{1} }}{dt}.$$

26 $$\begin{gathered} \frac{dL}{{dt}} = \left( {1 - \frac{{S_{h}^{1*} }}{{S_{h}^{1} }}} \right)(\Lambda_{\,h} + \mu_{\,h} C_{h}^{1} - \frac{{2\beta_{\,h} S_{h}^{1} I_{c}^{1} }}{{S_{h}^{1} + I_{c}^{1} }} - \phi_{\,h} S_{h}^{1} ) + \left( {1 - \frac{{I_{h}^{1*} }}{{I_{h}^{1} }}} \right)(\frac{{2\beta_{\,h} S_{h}^{1} I_{c}^{1} }}{{S_{h}^{1} + I_{c}^{1} }} - \delta_{\,h} I_{h}^{1} - \phi_{\,h} I_{h}^{1} ) + \left( {1 - \frac{{C_{h}^{1*} }}{{C_{h}^{1} }}} \right)(\delta_{\,h} I_{h}^{1} - \mu_{\,h} C_{h}^{1} - \phi_{\,h} C_{h}^{1} ) \hfill \\ \,\,\,\,\,\,\, + \left( {1 - \frac{{S_{c}^{1*} }}{{S_{h}^{1} }}} \right)(\Lambda_{\,c} + \mu_{\,c} I_{c}^{1} P_{\,c} - \frac{{2\beta_{\,c} S_{c}^{1} I_{c}^{1} }}{{S_{c}^{1} + I_{c}^{1} }} - \phi_{\,c} S_{c}^{1} ) + \left( {1 - \frac{{I_{c}^{1*} }}{{I_{h}^{1} }}} \right)(\frac{{2\beta_{\,c} S_{c}^{1} I_{c}^{1} }}{{S_{c}^{1} + I_{c}^{1} }} - \mu_{\,c} I_{c}^{1} P_{\,c} - \phi_{\,c} I_{c}^{1} ). \hfill \\ \end{gathered}$$

27 $$\begin{gathered} \frac{dL}{{dt}} = \Lambda_{\,h} + \mu_{\,h} C_{h}^{1} + \frac{{2\beta_{\,h} S_{h}^{1*} I_{c}^{1} }}{{S_{h}^{1} + I_{c}^{1} }} + S_{h}^{1*} \phi_{\,h} + \frac{{2\beta_{\,h} S_{h}^{1} I_{c}^{1} }}{{S_{h}^{1} + I_{c}^{1} }} + \delta_{\,h} I_{h}^{1*} + \phi_{\,h} I_{h}^{1*} + \mu_{\,h} C_{h}^{1*} + \phi_{\,h} C_{h}^{1*} + \Lambda_{\,c} + \mu_{\,c} I_{c}^{1} P_{\,c} \hfill \\ + \delta_{\,h} I_{h}^{1} + \frac{{2\beta_{\,c} S_{c}^{1} I_{c}^{1} S_{c}^{1*} }}{{S_{c}^{1} (S_{c}^{1} + I_{c}^{1} )}} + \phi_{\,c} S_{c}^{1*} + \frac{{2\beta_{\,c} S_{c}^{1} I_{c}^{1} }}{{S_{c}^{1} + I_{c}^{1} }} + \mu_{\,c} I_{c}^{1*} P_{\,c} + \phi_{\,c} I_{c}^{1*} - (\frac{{2\beta_{\,h} S_{h}^{1} I_{c}^{1} }}{{S_{c}^{1} + I_{c}^{1} }} + \phi_{\,h} S_{h}^{1} + \frac{{S_{h}^{1*} \Lambda_{\,h} }}{{S_{h}^{1} }} + \frac{{S_{h}^{1*} \mu_{\,h} C_{h}^{1} }}{{S_{h} }} \hfill \\ + \delta_{\,h} I_{h}^{1} + \phi_{\,h} I_{h}^{1} + \frac{{2\beta_{\,h} S_{h}^{1} I_{c}^{1} I_{h}^{1*} }}{{I_{h}^{1} (S_{h}^{1} + I_{c}^{1} )}} + \mu_{\,h} C_{h}^{1} + \phi_{\,h} C_{h}^{1} + \frac{{C_{h}^{1*} \delta_{\,h} I_{h}^{1} }}{{C_{h}^{1} }} + \frac{{2\beta_{\,c} S_{c}^{1} I_{c}^{1} }}{{S_{c}^{1} + I_{c}^{1} }} + \phi_{\,c} S_{c}^{1} \hfill \\ + \frac{{S_{c}^{1*} \Lambda_{\,c} }}{{S_{c}^{1} }} + \frac{{S_{c}^{1*} \mu_{\,c} I_{c}^{1} P_{\,c} }}{{S_{c}^{1} }} + \mu_{\,c} I_{c}^{1} P_{\,c} + \phi_{\,c} I_{c}^{1} + \frac{{2\beta_{\,c} S_{c}^{1} I_{c}^{1} I_{c}^{1*} }}{{I_{c}^{1} (S_{c}^{1} + I_{c}^{1} )}}). \hfill \\ \end{gathered}$$

28 $$\frac{dL}{{dt}} = C - D.$$

where

29 $$\begin{gathered} A = \Lambda_{\,h} + \mu_{\,h} C_{h}^{1} + \frac{{2\beta_{\,h} S_{h}^{1*} I_{c}^{1} }}{{S_{h}^{1} + I_{c}^{1} }} + S_{h}^{1*} \phi_{\,h} + \frac{{2\beta_{\,h} S_{h}^{1} I_{c}^{1} }}{{S_{h}^{1} + I_{c}^{1} }} + \delta_{\,h} I_{h}^{1*} + \phi_{\,h} I_{h}^{1*} + \mu_{\,h} C_{h}^{1*} + \phi_{\,h} C_{h}^{1*} + \Lambda_{\,c} + \mu_{\,c} I_{c}^{1} P_{\,c} \hfill \\ \,\,\,\,\,\,\,\,\, + \delta_{\,h} I_{h}^{1} + \frac{{2\beta_{\,c} S_{c}^{1} I_{c}^{1} S_{c}^{1*} }}{{S_{h}^{1} (S_{c}^{1} + I_{c}^{1} )}} + \phi_{\,c} S_{c}^{1*} + \frac{{2\beta_{\,c} S_{c}^{1} I_{c}^{1} }}{{S_{c} + I_{c} }} + \mu_{\,c} I_{c}^{1*} P_{\,c} + \phi_{\,c} I_{c}^{1*} . \hfill \\ B = \frac{{2\beta_{\,h} S_{h}^{1} I_{c}^{1} }}{{S_{h}^{1} + I_{c}^{1} }} + \phi_{\,h} S_{h}^{1} + \frac{{S_{h}^{1*} \Lambda_{\,h} }}{{S_{h}^{1} }} + \frac{{S_{h}^{1*} \mu_{\,h} C_{h}^{1} }}{{S_{h}^{1} }} + \delta_{\,h} I_{h}^{1} + \phi_{\,h} I_{h}^{1} + \frac{{2\beta_{\,h} S_{h}^{1} I_{c}^{1} I_{h}^{1*} }}{{I_{h}^{1} (S_{h}^{1} + I_{c}^{1} )}} + \mu_{\,h} C_{h}^{1} + \phi_{\,h} C_{h}^{1} + \frac{{C_{h}^{1*} \delta_{\,h} I_{h}^{1} }}{{C_{h}^{1} }} \hfill \\ \,\,\,\,\,\,\,\, + \frac{{2\beta_{\,c} S_{c}^{1} I_{c}^{1} }}{{S_{c}^{1} + I_{c}^{1} }} + \phi_{\,c} S_{c}^{1} + \frac{{S_{c}^{1*} \Lambda_{\,c} }}{{S_{c}^{1} }} + \frac{{S_{c}^{1*} \mu_{\,c} I_{c}^{1} P_{\,c} }}{{S_{c}^{1} }} + \mu_{\,c} I_{c}^{1} P_{\,c} + \phi_{\,c} I_{c}^{1} + \frac{{2\beta_{\,c} S_{c}^{1} I_{c}^{1} I_{c}^{1*} }}{{I_{c}^{1} (S_{c}^{1} + I_{c}^{1} )}}. \hfill \\ \end{gathered}$$

Thus if $$A < B\,\,then\,\frac{dL}{{dt}} < 0$$.

Noting that: $\frac{dL}{{dt}} = 0$ if and only if $S_{h}^{1*} = S_{h}^{1} ,\,I_{h}^{1*} = I_{h}^{1} ,\,\,C_{h}^{1*} = C_{h}^{1} ,\,S_{c}^{1*} = S_{c}^{1} ,\,I_{c}^{1*} = I_{c}^{1} .$ Therefore, the largest compact invariant set in $\left\{ {\left( {S_{h}^{1*} ,I_{h}^{1*} ,C_{h}^{1*} ,S_{c}^{1*} ,I_{c}^{1*} } \right) \in R^{5} :\frac{dL}{{dt}} = 0} \right\}$ is the singleton $E^{*}$, by invariance principle, it implies that $E^{*}$ is GAS in $R^{5}$ if $A < B$. This implies that $R_{0} > 1$ ^38^.

## Sensitivity analysis

Model (1) parameters were analyzed based on sensitivity analysis to detect parameters with high transmission influences^39^. We analyze reproduction numbers to see if treating infection and mortality could lead to effective control of animals and vectors. Using the following relation, we determine the most sensitive parameter:

$P_{q} = \frac{q}{{R_{0} }} \times \frac{{\partial R_{0} }}{\partial q}$, where *q* are parameter and $R_{0}$ is the reproductive number.

If $P_{q} < 1$, the parameter $q$ becomes influential in controlling the disease.

If $P_{q} > 1$, then $q$ assumes a role in disease control.

The sensitivity indices of $R_{\,0}$ with corresponding parameters, analytical and numerical values are given in Table 2.

**Table 2:** Sensitivity indices of $R_{\,0}$.

No.	Parameters	Analytical values	Numerical values
1	$\beta_{\,c}$	1	1
2	$P_{\,c}$	$- \frac{{P_{c} \mu_{c} }}{{P_{c} \mu_{c} + \phi_{c} }}$	$-0.014778325123152707$
3	$\mu_{\,c}$	$- \frac{{\mu_{c}^{2} }}{{P_{c} \mu_{c} + \phi_{c} }}$	$-12.31527093596059$
4	$\phi_{\,c}$	$- \frac{{\mu_{c} \phi_{c} }}{{P_{c} \mu_{c} + \phi_{c} }}$	$-0.49261083743842365$

The sensitivity analysis presented in Table 2 indicates that certain parameters demonstrate a greater sensitivity to the reproduction number than others. Infection spread is reduced by these three parameters $P_{\,c}$**, **$\mu_{\,c}$ and $\phi_{\,c}$. $\beta_{\,c}$, on the other hand, increases the spread of the infection when it gets high.

**Figure 2:**
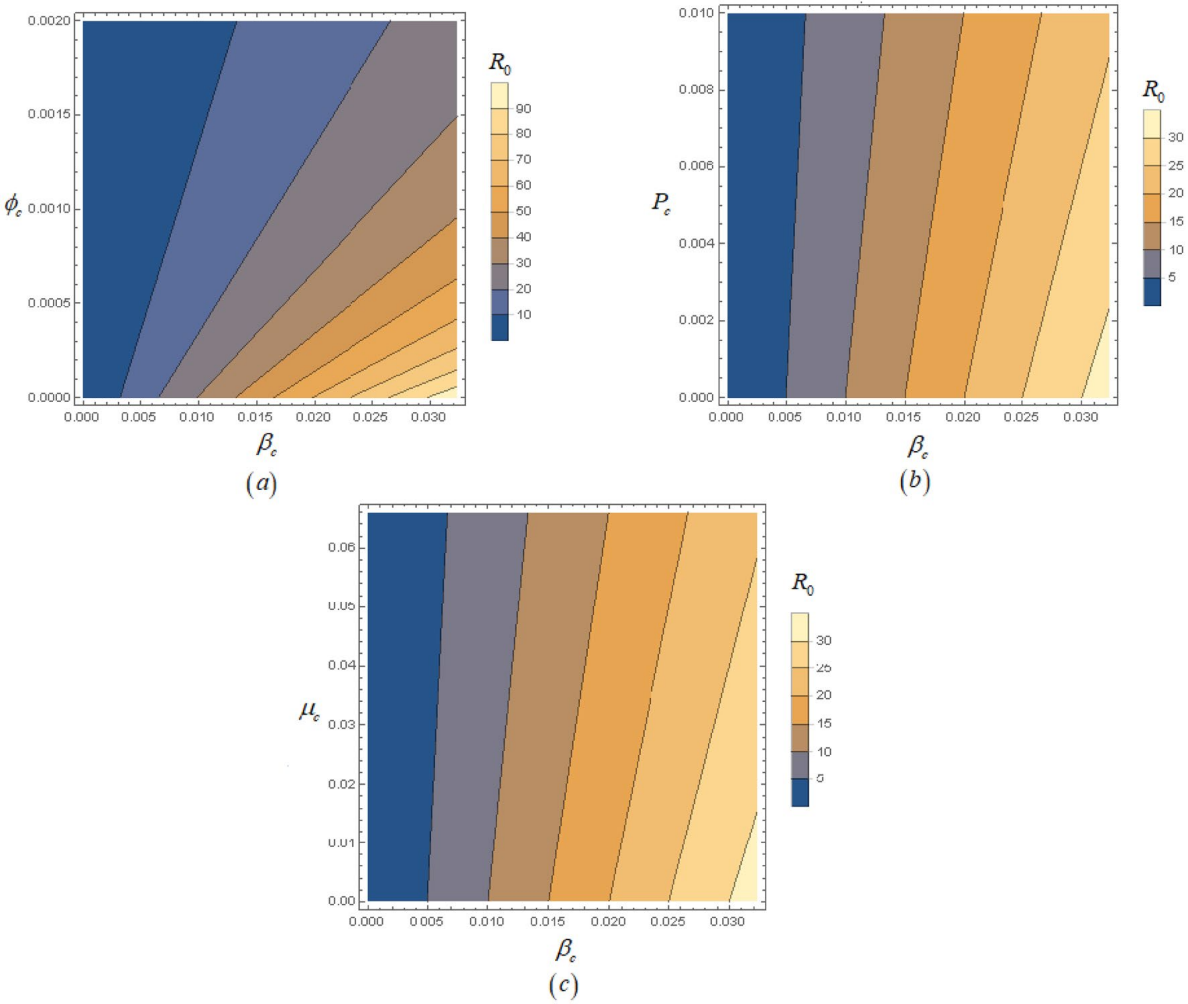
Counterplot of $$R_{\,0}$$.

Based on the parameter values given in Table 3, we have plotted the reproduction number as a function as a function of $\beta_{\,c}$ and $\phi_{\,c}$,$\beta_{\,c}$ and $P_{\,c}$, $\beta_{\,c}$ and $\mu_{\,c}$. This plot demonstrates the relationship between the primary and secondary parameters, as well as their sensitivity effects. In Fig. 2a, we observe the impact of the effectual contact rate and natural mortality rate on the reproduction number. It is evident that these two parameters may decrease to less than unity. Figure 2b illustrates the effect of transmission rates $\beta_{\,c}$ and $P_{\,c}$. We can clearly see that these parameters significantly influence whether the value of the basic reproduction number is reduced or increased. Figure 2c shows the relation between $\beta_{\,c}$ and $\mu_{\,c}$ to reproduction number. As it can be that $\beta_{\,c}$ is directly and $\mu_{\,c}$ is inversely proportional to the reproduction number.

**Table 3:** Parameter values of Toxoplasmosis disease model.

Parameters	Estimated values	Reference
$\Lambda_{h}$	0.0001	Assumed
$\mu_{h}$	0.233	^7^
$\beta_{h}$	0.0206	^7^
$\phi_{h}$	0.001	Assumed
$\delta_{h}$	0.000232	^18^
$\Lambda_{c}$	0.0003	Assumed
$\mu_{c}$	0.066	^18^
$\beta_{c}$	0.0232	Assumed
$\phi_{c}$	0.002	Assumed
$P_{c}$	0.01	^18^

## Numerical simulation

Following are numerical simulations of both human and cat populations affected by Toxoplasmosis. The state equations are solved over a period of time by using the Runge–Kutta forth order scheme by using MATLAB sowfter.

## Results and discussion

The numerical results of the model (1) for various parameter values are presented. The values of parameters are given in Table 3. The initial conditions for the simulations and analyses of model (1) are: $S_{h}^{1} (0) = 100$, $I_{h}^{1} (0) = 60$, $C_{h}^{1} (0) = 30$, $S_{c}^{1} (0) = 100$ and $I_{c}^{1} (0) = 30$.

**Figure 3:**
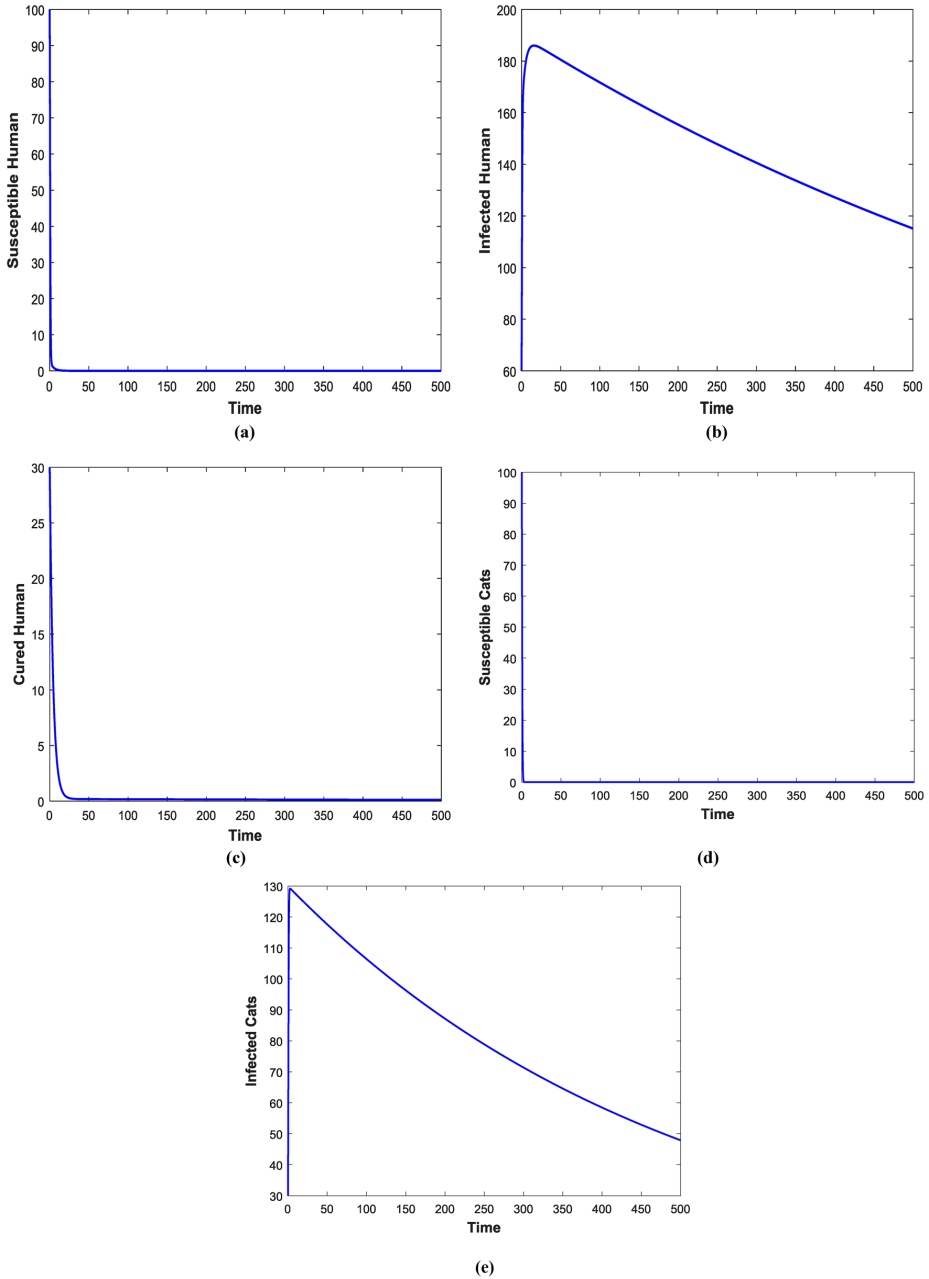
Shows the dynamics of Toxoplasmosis in the human and cats population with bilinear incident rate.

**Figure 4:**
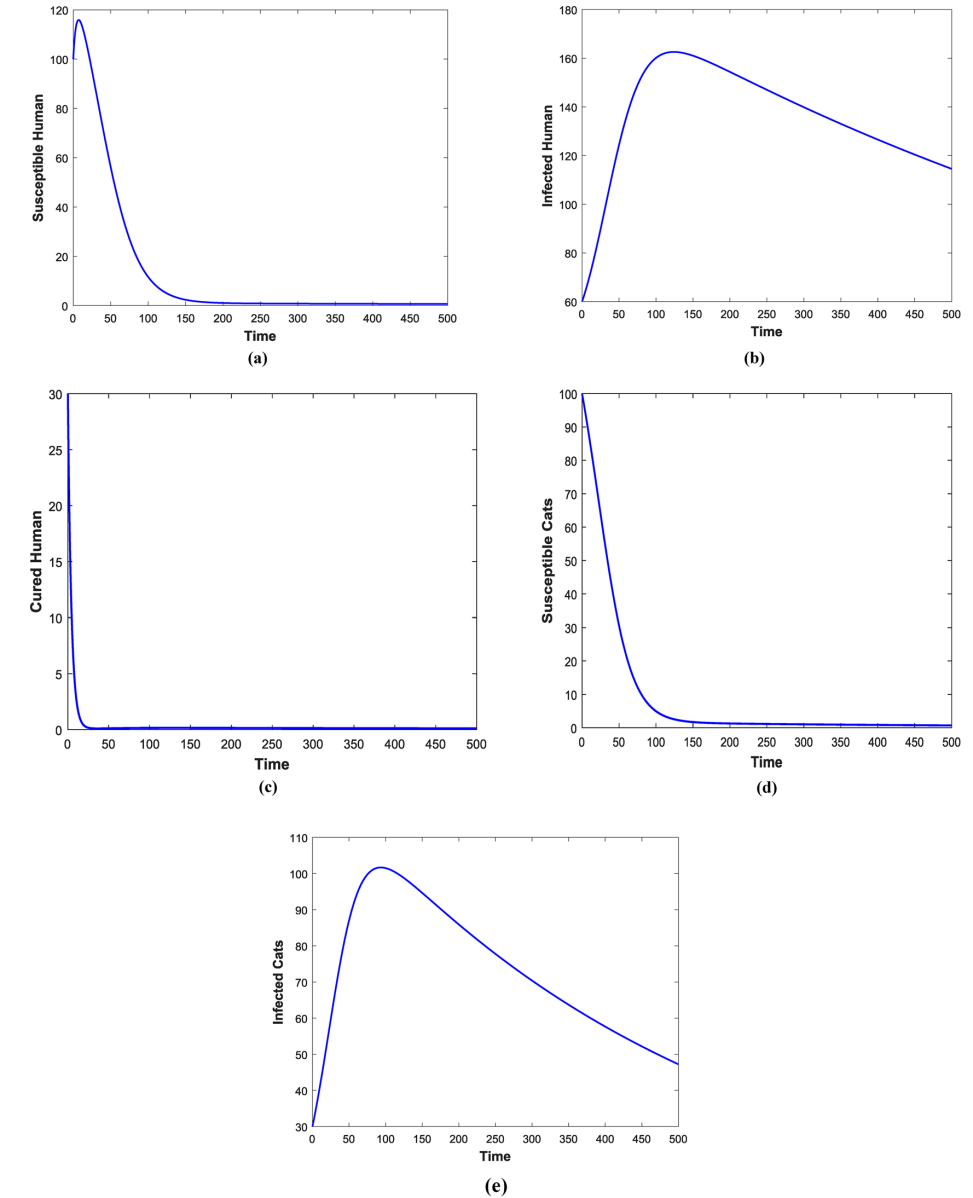
Shows the dynamics of Toxoplasmosis in the human and cats population with harmonic mean type incident rate.

**Figure 5:**
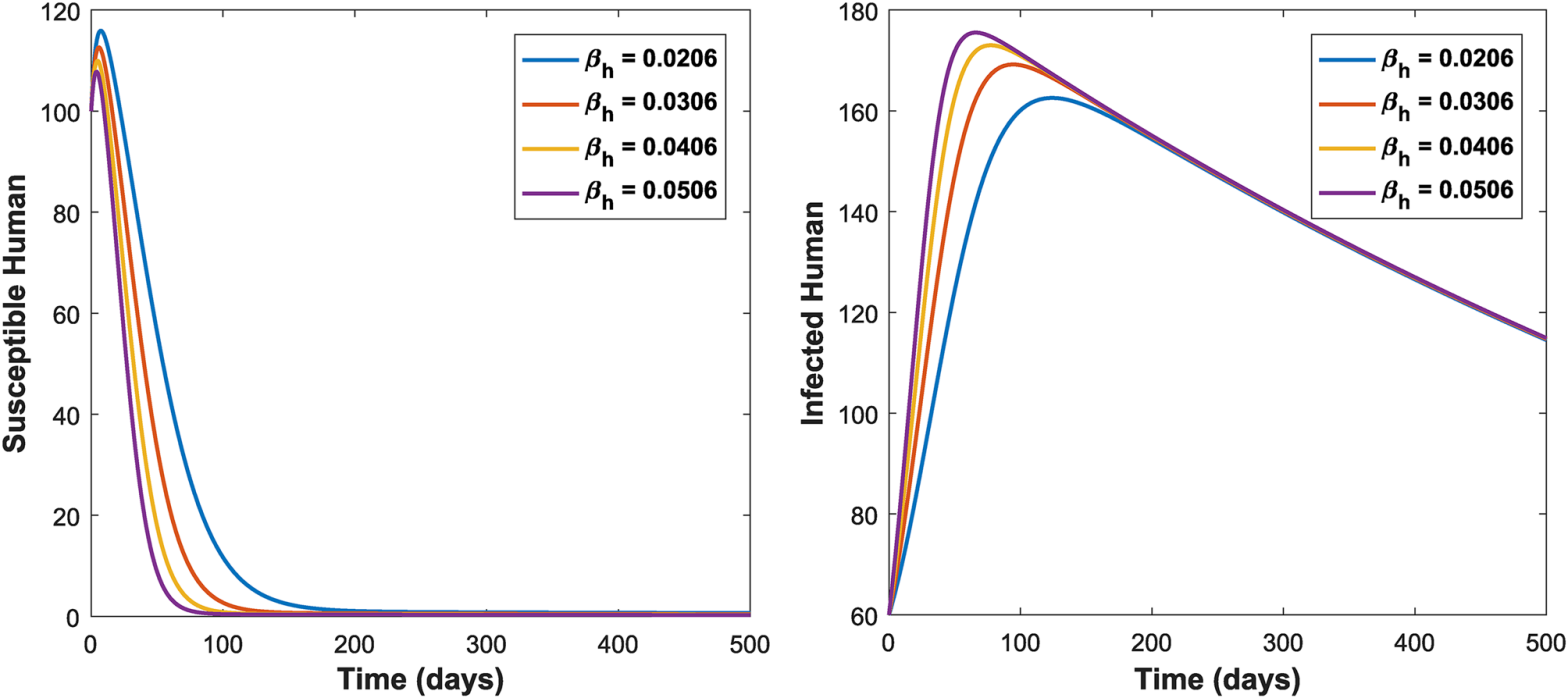
Shows the behavior of susceptible and infected human individual under the influence of $\beta_{h}$. When $\Lambda_{c} = 0.0003$, $\mu_{c} = 0.066$, $\beta_{h} = 0.0206$, $\phi_{h} = 0.001$, $\beta_{c} = 0.0232$, $\phi_{c} = 0.002$, $P_{c} = 0.01$, $\Lambda_{h} = 0.0001$, $\mu_{h} = 0.233$, $\delta_{h} = 0.000323$.

**Figure 6:**
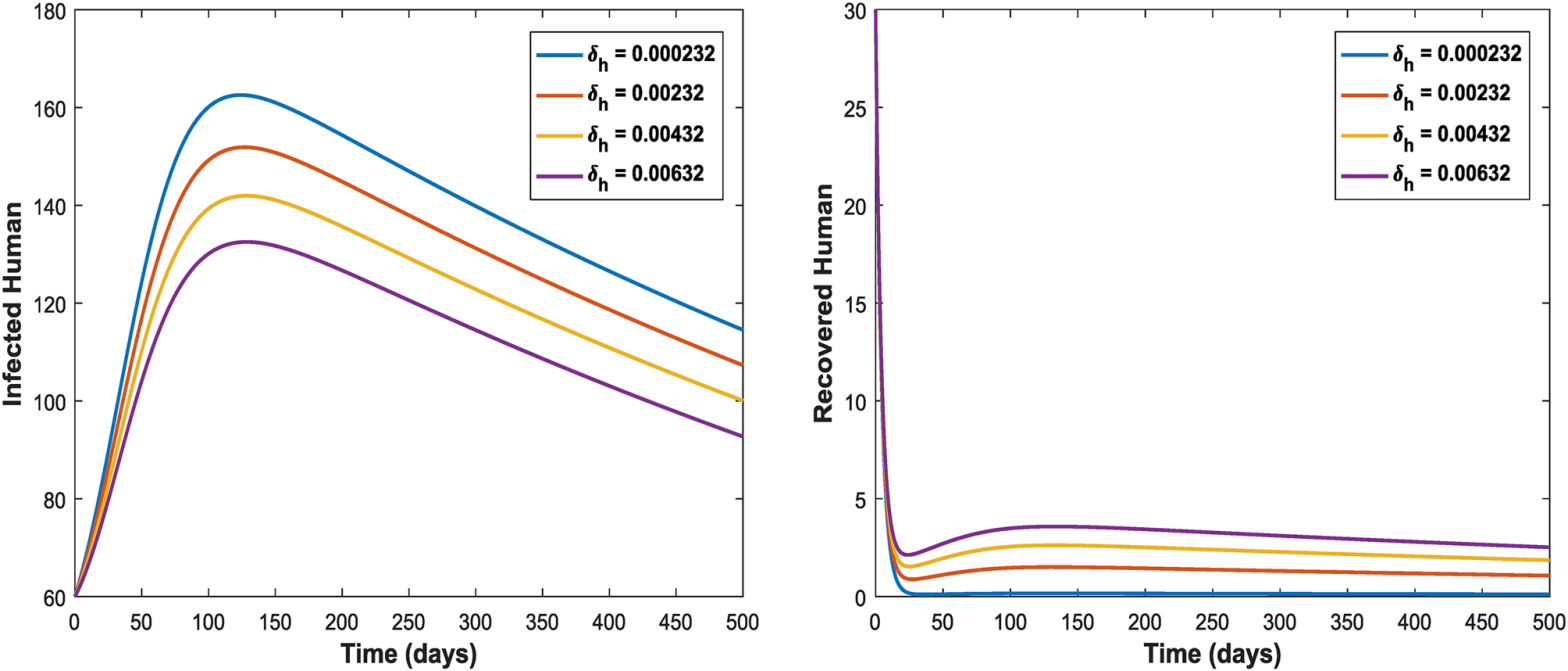
Shows the behavior of infected and recovered human individual with the effect of $\delta_{h}$. When $\Lambda_{c} = 0.0003$, $\mu_{c} = 0.066$, $\beta_{h} = 0.0206$, $\phi_{h} = 0.001$, $\beta_{c} = 0.0232$, $\phi_{c} = 0.002$, $P_{c} = 0.01$, $\Lambda_{h} = 0.0001$, $\mu_{h} = 0.233$, $\delta_{h} = 0.000323$.

**Figure 7:**
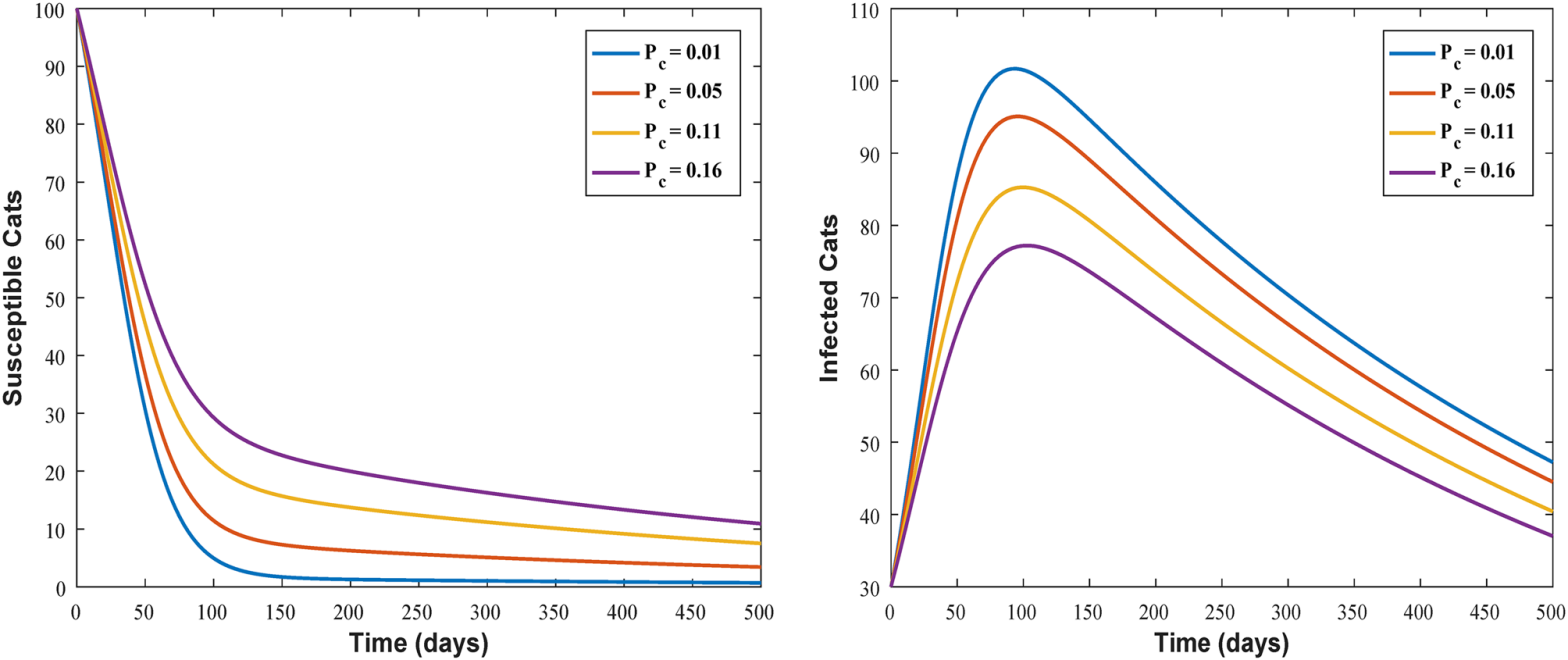
Shows the behavior of susceptible and infected cat individual with the effect of $P_{c}$. When $\Lambda_{c} = 0.0003$, $\mu_{c} = 0.066$, $\beta_{h} = 0.0206$, $\phi_{h} = 0.001$, $\beta_{c} = 0.0232$, $\phi_{c} = 0.002$, $P_{c} = 0.01$, $\Lambda_{h} = 0.0001$, $\mu_{h} = 0.233$, $\delta_{h} = 0.000323$.

**Figure 8:**
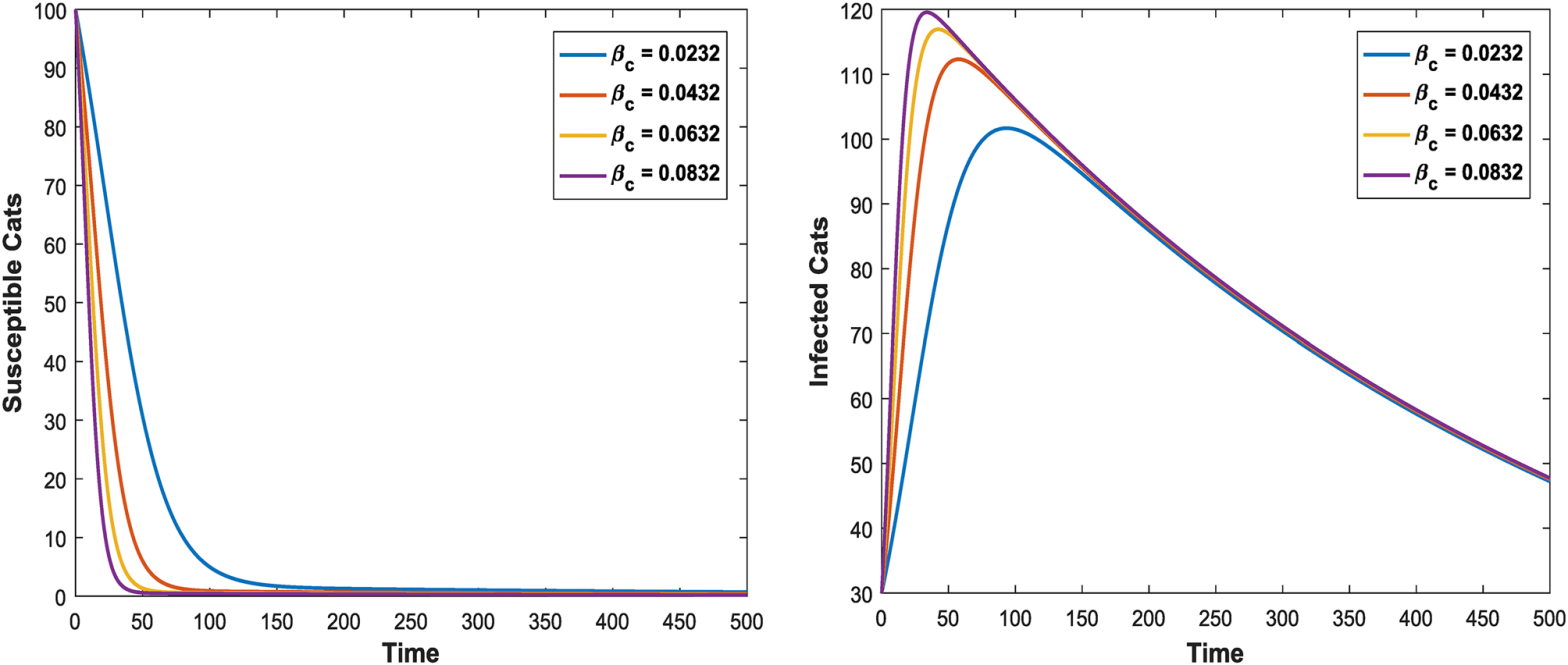
Shows the behavior of susceptible and infected cat individual with the effect of $\beta_{c}$ when $\Lambda_{c} = 0.0003$, $\mu_{c} = 0.066$, $\beta_{h} = 0.0206$, $\phi_{h} = 0.001$, $\beta_{c} = 0.0232$, $\phi_{c} = 0.002$, $P_{c} = 0.01$, $\Lambda_{h} = 0.0001$, $\mu_{h} = 0.233$, $\delta_{h} = 0.000323$.

**Figure 9:**
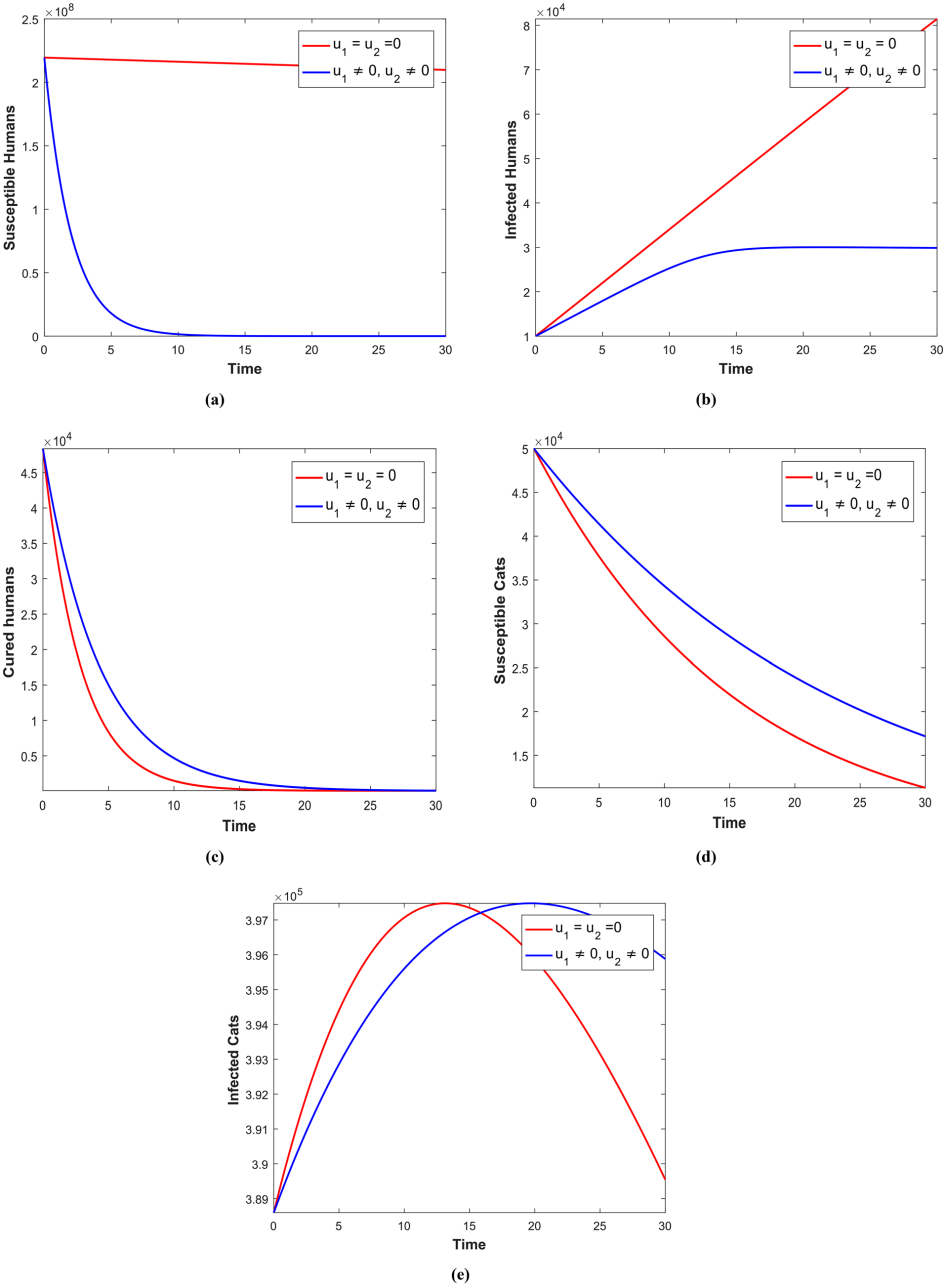
Simulation depicting the optimal outcomes for susceptibile human, infected human, recovered human, susceptible cats, and infected cats achieved through the utilization of $u_{1}$ and $u_{2}$.

Figures 3 and 4 shows the impact of bilinear and harmonic means type incident rate on the dynamic of Toxoplasmosis in the human and cat population. From Fig. 3, it can been seen that by using bilinear incident rate the disease suddenly spread in the population while Fig. 4 shows that the spread gradually in the human and cats population which is more realistic to real word problems as compare to bilinear incident rate. Figures 5 and 6 illustrate the impact of contact rates on the susceptible and infected populations of human. These figures illustrate that when infected individuals come into contact with susceptible individuals, susceptible individuals will attract the disease then susceptible individuals eventually decrease, while infected ones increase which supports the first two equations of model (1). Figures 7 and 8 depict the impact of $\delta_{h}$ on the infected and cured populations of humans. These figures indicate that when the value of $\delta_{h}$ increases, the infected individuals who recover from the infectious disease also increases over time. Consequently, Eventually, infected individuals decrease, but icured ones increase which is evident from 2nd and 3rd eqution of model (1). As shown in Figs. 9 and 10, $P_{c}$ has a major impact on the susceptible and infected cats. As the probability of an infected cat giving birth to a susceptible one increases, the data presented in these figures demonstrates. the susceptible cats in the population also increases over time, while the number of infected cats also increases which supports 4th and 5th equations of model (1). Figures 11 and 12 illustrate the impact of contact rates on the susceptible and infected populations of cat. These figures demonstrate that when an infected individual comes into contact with susceptible cats, the susceptible cats decreases over time, while the infected cats increases.

## Optimal control model of toxoplasmosis

This section addresses the optimal control stratiges of toxoplasmosis. The optimal control model (30) reduces infected numbers of population by revisiting the Toxoplasmosis model (1). The spread of toxoplasmosis disease is reduced by using two control variables, $u_{1}$ and $u_{2}$. $u_{1}$ is the vaccination of toxoplasmosis for both population and $u_{2}$ represent the awarence about the toxoplasmosis disease in the population. The following nonlinear system of ordinary differential equations describes the model with control.

30 $$\left. \begin{gathered} \frac{{dS_{h}^{1} }}{dt} = \Lambda_{h} + \mu_{h} C_{h}^{1} - \left( {1 - u_{1} } \right)\frac{{2\beta_{h} S_{h}^{1} I_{c}^{1} }}{{S_{h}^{1} + I_{c}^{1} }} - \left( {u_{2} + \phi_{h} } \right)S_{h}^{1} , \hfill \\ \frac{{dI_{h}^{1} }}{dt} = \left( {1 - u_{1} } \right)\frac{{2\beta_{h} S_{h}^{1} I_{c}^{1} }}{{S_{h}^{1} + I_{c}^{1} }} + \delta_{h} I_{h}^{1} - \phi_{h} I_{h}^{1} , \hfill \\ \frac{{dC_{h}^{1} }}{dt} = \delta_{h} I_{h}^{1} - \mu_{h} C_{h}^{1} - \phi_{h} C_{h}^{1} , \hfill \\ \frac{{dS_{c}^{1} }}{dt} = \Lambda_{c} + \mu_{h} I_{c}^{1} P_{c} - \left( {1 - u_{1} } \right)\frac{{2\beta_{c} S_{c}^{1} I_{c}^{1} }}{{S_{c}^{1} + I_{c}^{1} }} - \phi_{c} S_{c}^{1} , \hfill \\ \frac{{dI_{c}^{1} }}{dt} = \left( {1 - u_{1} } \right)\frac{{2\beta_{c} S_{c}^{1} I_{c}^{1} }}{{S_{c}^{1} + I_{c}^{1} }} - \mu_{h} I_{c}^{1} P_{c} - \phi_{c} I_{c}^{1} . \hfill \\ \end{gathered} \right\}.$$

Taking into account initial condition if model (1).

An integrated model identifies the best level of intervention strategy to mitigate disease transmission using control measures. Costs associated with controlling spreads and the costs associated with implementing these controls must both be assessed. A differential Eq. (30) is used to minimize the control variables $u_{1}$ and $u_{2}$, where the objective function is formulated as follows:

31 $$J(u_{1} ,u_{2} ) = \int\limits_{0}^{{t_{f} }} {\left[ {A_{1} I_{h} + A_{2} I_{c} + \frac{1}{2}\left( {A_{3} u_{1}^{2} + A_{4} u_{2}^{2} } \right)} \right]} dt$$

In accordance with (30). In the objective functional, weight constants $A_{1} ,A_{2} ,A_{3}$ and $A_{3}$ represent an infected population. The terms $\frac{{A_{3} u_{1}^{2} }}{2}$ and $\frac{{A_{4} u_{3}^{2} }}{2}$ refer to the costs associated with the corresponding interventions. The corresponding control function is assumed to be proportional to the square of the costs. In order to solve the optimal control problem, we must find the optimal control functions $u_{1}^{*}$ and $u_{2}^{*}$ such that

32 $$J\left( {u_{1}^{*} ,u_{2}^{*} } \right) = \min \left\{ {J\left( {u_{1} ,u_{2} } \right),\left( {u_{1} ,u_{2} } \right) \in U} \right\},$$

The control set is defined by the system (30) as follows:

33 $$U = \left\{ {\left. {\left( {u_{1} ,u_{2} } \right)} \right|u_{i} \left( t \right)\,\,is\,Lebesgue\,\,measure\,\,on\,[0,1],i = 1,2} \right\}.$$

Making use of Pontryagin's maximum principle, the objective functional and state variables $u$ together can be minimized or maximized in relation to the Hamiltonian H. This optimal control problem is derived using the Pontryagin maximum principle. For optimal control problems (30) to (32), The Lagrangian and Hamiltonian must first be determined. This is how you express lagrangians for optimal problems:

34 $$L = A_{1} I_{h}^{1} + A_{2} I_{c}^{1} + \frac{1}{2}\left( {A_{3} u_{1}^{2} + A_{4} u_{2}^{2} } \right)$$

The Hamiltonian for the control problem is given by a minimal value of the Lagrangian:

35 $$H = L\left( {C,u_{1} ,u_{2} } \right) + \lambda_{1} \frac{{dS_{h}^{1} }}{dt} + \lambda_{2} \frac{{dI_{h}^{1} }}{dt} + \lambda_{3} \frac{{dC_{h}^{1} }}{dt} + \lambda_{4} \frac{{dS_{c}^{1} }}{dt} + \lambda_{5} \frac{{dI_{c}^{1} }}{dt}$$

where $\lambda_{i} ,\,i = 1,2,...5$ are the adjoint variables. In order to derive the optimality system for system (30), we need to apply Lukes'^40^ results and prove the existence of an optimal control scheme. In fact, for the existence of this optimal control, we use the results in Lukes^40^. A control variable and a state variable have nonnegative values. This minimizing problem allows the objective functional $L$ to satisfy the necessary convexity. The set of all the control variables $\left( {u_{1} ,u_{2} } \right) \in U$ is also convex and closed by definition. Moreover, the integrand in Eq. (30), $A_{1} I_{h}^{1} + A_{2} I_{c}^{1} + \frac{1}{2}\left( {A_{3} u_{1}^{2} + A_{4} u_{2}^{2} } \right)$, is convex in the control set U because the optimal system is bounded; therefore, an optimal control exist. Also, we can easily see that there exists a constant $\rho > 0$ and positive numbers $\Xi_{1} ,\Xi_{2}$ such that

36 $$J\left( {u_{1} ,u_{2} } \right) \ge \Xi_{1} \left( {\left| {u_{1} } \right|^{2} + \left| {u_{2} } \right|^{2} } \right)^{\frac{l}{2}} - \Xi_{2} ,$$

As the state variables are bounded, we can conclude that an optimal control exists.

### Theorem:

*There exists an optimal control* $u^{*} = \left( {u_{1}^{*} ,u_{2}^{*} } \right) \in U$ *such that*

37 $$J\left( {u_{1}^{*} ,u_{2}^{*} } \right) = \mathop {\min }\limits_{{\left( {u_{1} ,u_{2} } \right)\, \in \,U}} J\left( {u_{1} ,u_{2} } \right)$$

Depending on initial conditions (30).

The Hamiltonian (35) is subject to Pontryagin's maximum principle^41^, which implies that there is a nontrivial vector function $\lambda = \left( {\lambda_{1} ,\lambda_{2} ,......,\lambda_{n} } \right)$ that meets the following conditions if $\left( {x,u} \right)$ is an optimal response to the optimal control problem:

38 $$\left. \begin{gathered} x^{\prime} = \frac{{\partial H\left( {t,x,u,\lambda } \right)}}{\partial \lambda }, \hfill \\ 0 = \frac{{\partial H\left( {t,x,u,\lambda } \right)}}{\partial u}, \hfill \\ \lambda^{\prime} = \frac{{\partial H\left( {t,x,u,\lambda } \right)}}{\partial x}. \hfill \\ \end{gathered} \right\}$$

The Hamiltonian H is then treated as necessary in (38).

### Theorem:

*Let *$S_{h}^{1*} ,I_{h}^{1*} ,C_{h}^{{1*}{}} ,S_{c}^{1*} \,\,{\text{and}}\;\; \, I_{c}^{1*}$* be optimal state solutions with associated optimal control variables *$u_{1}^{*} ,u_{2}^{*}$* for the optimal control problems *(30)* and *(31).* Then there exist adjoint variables *$\lambda \,_{i} {\text{ for }}\,i = 1,2,3....5$* satisfying*

39 $$\left. \begin{gathered} \lambda_{1} = \frac{{2I_{c}^{1} S_{h}^{1} \left( { - 1 + u_{1} } \right)\beta_{h} }}{{I_{c}^{1} + S_{h}^{1} }} + \Lambda_{h} + C_{h}^{1} \mu_{h} - S_{h}^{1} \left( {u_{2} + \phi_{h} } \right), \hfill \\ \lambda_{2} = - \frac{{2I_{c}^{1} S_{h}^{1} \left( { - 1 + u_{1} } \right)\beta_{h} }}{{I_{c}^{1} + S_{h}^{1} }} + I_{h}^{1} \left( {\delta_{h} - \phi_{h} } \right), \hfill \\ \lambda_{3} = - C_{h}^{1} \mu_{h} + I_{h}^{1} \left( {\delta_{h} - \phi_{h} } \right), \hfill \\ \lambda_{4} = \frac{{2I_{c}^{1} S_{c}^{1} \left( { - 1 + u_{1} } \right)\beta_{c} }}{{I_{c}^{1} + S_{c}^{1} }} + \Lambda_{c} + I_{c}^{1} P_{c} \mu_{h} - \phi_{c} S_{c}^{1} , \hfill \\ \lambda_{5} = I_{c}^{1} \left( { - \frac{{2S_{c}^{1} \left( { - 1 + u_{1} } \right)\beta_{c} }}{{I_{c}^{1} + S_{c}^{1} }} - P_{c} \mu_{h} - \phi_{c} } \right). \hfill \\ \end{gathered} \right\}$$

A boundary condition or a transversality condition

$$\lambda_{i} \left( T \right){ = 0 , }\,i = 1,2,3....5$$

Furthermore, the control functions $u_{1}^{*} \,{\text{and }}u_{2}^{*}$ are given by

40 $$\left. \begin{gathered} u_{1}^{*} = max\left\{ {\min \left( {\frac{{2I_{c}^{1} (S_{c}^{1} S_{h}^{1} \left( {\beta_{h} \left( { - \lambda_{1} + \lambda_{2} } \right) + \beta_{c} \left( { - \lambda_{4} + \lambda_{5} } \right)} \right) + I_{c}^{1} \left( {S_{h}^{1} \beta_{h} \left( { - \lambda_{1} + \lambda_{2} } \right) + S_{c}^{1} \beta_{c} \left( { - \lambda_{4} + \lambda_{5} } \right)} \right)}}{{C_{3} \left( {I_{c}^{1} + S_{c}^{1} } \right)\left( {I_{c}^{1} + S_{h}^{1} } \right)}},1} \right),0} \right\}, \hfill \\ u_{2}^{*} = max\left\{ {\min \left( {\frac{{S_{h}^{1} \lambda_{1} + S_{c}^{1} \lambda_{4} }}{{C_{4} }},1} \right),0} \right\}. \hfill \\ \end{gathered} \right\}.$$

Hence, the optimal control pair is characterized.

### Numerical simulation of optimal control model

Using numerical simulations, we examine how vaccination and awareness can reduse the spread of disease by conducting numerical simulations. At times $t = 0$ and t = $t = t_{f}$, the boundary conditions for the optimality system are separated. As shown in Fig. 13a, in the absence of control measure means there is no awareness of toxoplasmosis disease, a higher number of susceptible individuals exists. However, when vaccines and awareness are introduced into the population, the number of susceptible people decreases rapidly. As can be seen in Fig. 13b, in the absence of control measures, there is a higher number of human individuals infected with toxoplasmosis. However, when vaccination and education are introduced into the population regarding toxoplasmosis, the number of infected individuals decreases rapidly. As can be seen in Fig. 13c, when there is no vaccination and awareness about toxoplasmosis disease, human individuals recover from the disease at a low rate; however, when vaccination and awareness about the disease are introduced into the population, the number of recovered humans increases rapidly. As shown in Fig. 13d, the number of susceptible cats is higher in the absence of controlling measures, and in the presence of controlling measures, the population of susceptible cats rapidly decreases. In Fig. 13e it can be seen that in the absence of control measure the number of infected cat individuals is higher but in the presence the number infected population is decreased rapiadly.

## Conclusion

In this research, a mathematical model was developed to describe the dynamics of the transmission of Toxoplasmosis among humans and cats. Humans population are classified into three categories susceptible, infected, and cured, and cats population are divided into susceptibles and infections. Disease extinction and endemic equilibrium points are presented. To find reproduction number, the next generation matrix method is used, and it is found that reproduction number completely depends on the parameters of the cat population. The stability of endemic and disease-free equilibrium is analyzed by using $R_{0}$. The DFE is stable both globally and locally when $R_{0} < 1$. When $R_{0} > 1$, both globally and locally, the endemic equilibrium is stable. Sensitivity analysis of reproduction number is conducted. Numerical stimulation is also used to assess how each parameter affects expansion and the management of Toxoplasmosis disease. The disease is controlled in human by the treatment parameter in response to numerical stimulation, while the disease is exacerbated by the contact rate. Similary the disease also increases in the cat population wuth the increase of $\beta_{c}$. The optimal control stratiges is investigated to control the spread of disese. It is observed that the the control measures like vaccination of disease and the education about the disease reduced the spread of disease dramatically. The outcomes of this study can be used by various departments such as public health departments, veterinary departments, and medical research institutions. The model can provide insights into the dynamics of the disease and help identify potential control measures. The information obtained from the model can be used to inform policy decisions related to disease prevention and control, as well as to design effective intervention strategies. Furthermore, the model's predictions can assist in prioritizing limited resources towards controlling and treating the disease, and thus potentially benefit human and cats health. In the future, this research work can be extended by incorporating exposed compartment, vertical transmission of the disease. This work can also be extended to fractional model.

## Data Availability

Data used in this work is available from the corresponding author base on a reasonable request.
